# Supplementary material for: Deregulation of Cholesterol Homeostasis by a Nuclear Hormone Receptor Crosstalk in Advanced Prostate Cancer
Source: Cancers (Basel). 2022 Jun 24;14(13):3110. doi: 10.3390/cancers14133110 (PMC9265016; doi:10.3390/cancers14133110)
Supplement: Supplementary file 1 [file cancers-14-03110-s001.zip › cancers-1791879-supplementary.pdf]

Article

Supplementary Materials: Deregulation of Cholesterol Homeostasis by a Nuclear Hormone Receptor Crosstalk in Advanced Prostate Cancer

Nianxin Yang, Yatian Yang, Zenghong Huang and Hong-Wu Chen*

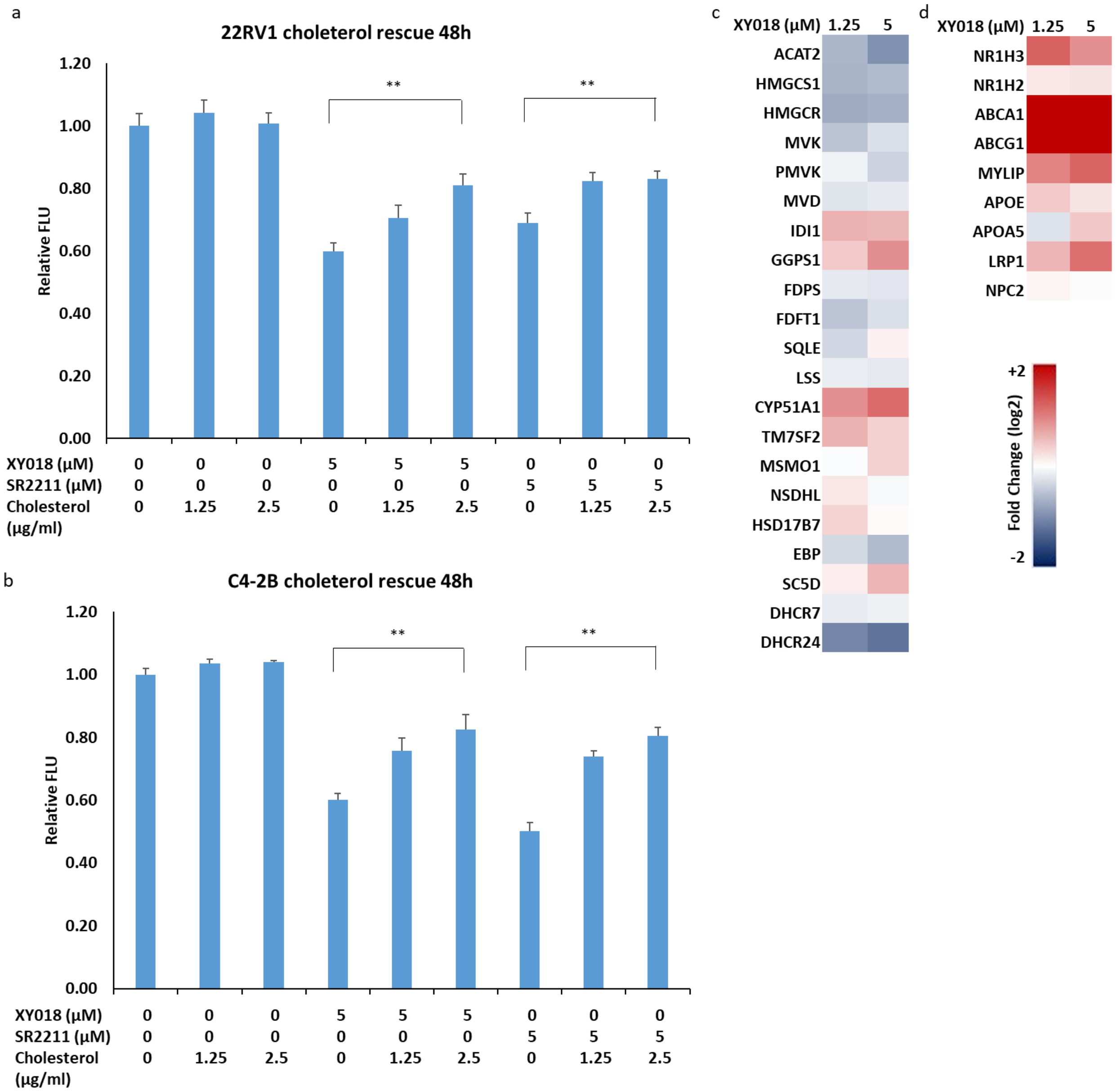

Figure S1. (**a and b**). Exogenous cholesterol rescued growth inhibition by RORγ antagonists in mCRPC cells. Cell viability was measured by CellTiter-Glo Assay of 22Rv1 (**a**) and C4-2B (**b**) cells treated with indicated concentration of XY018, SR2211, and cholesterol for 48 h. Data are shown as mean ± s.d. n = 3. Student's t test. $*p < 0.05$, $**p < 0.01$. (**c and d**). RORγ antagonist inhibit the expression of cholesterol biosynthesis genes and enhances the expression of cholesterol efflux genes in androgen-responsive LNCaP cells. Heat maps display mRNA expression of 21 cholesterol biosynthesis genes (**c**) or 7 cholesterol efflux genes (**d**) measured by qRT-PCR in LNCaP cells treated with indicated concentrations of XY018 for 48 h, as compared to vehicle (DMSO). n = 3.

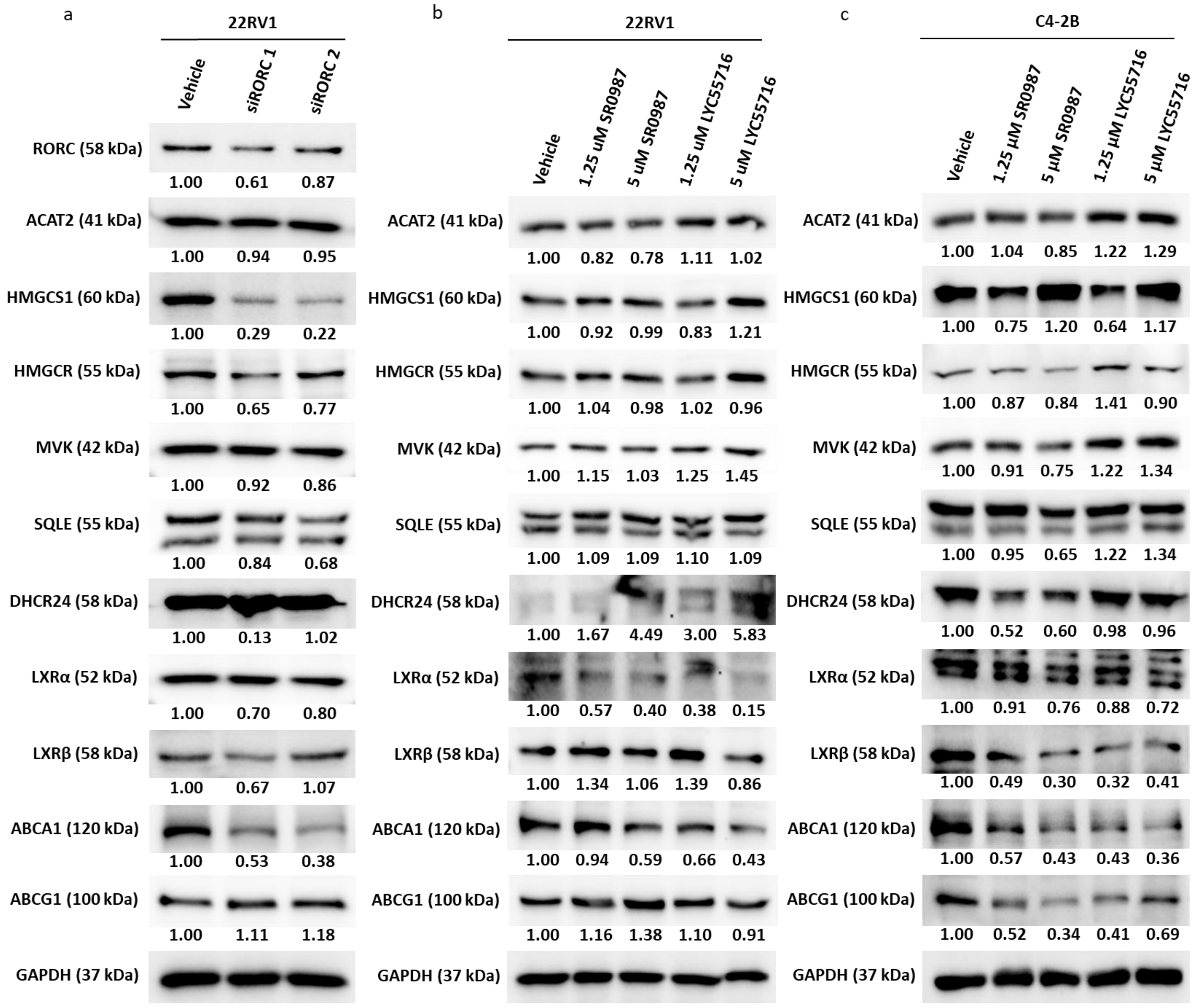

Figure S2. (**a**). Knockdown of RORγ inhibits the expression of key cholesterol biosynthesis proteins and enhances the expression of key cholesterol efflux proteins. Immunoblotting of RORγ and proteins involved in cholesterol biosynthesis and cholesterol efflux pathways in 22Rv1 cells treated with indicated siRNAs for 72 h. (**b and c**). RORγ agonist treatment show effects oppositive to that of its antagonists on the protein expressions in mCRPC cells. Immunoblotting of proteins involved in cholesterol biosynthesis and cholesterol efflux pathways in 22Rv1 (**b**) and C4-2B (**c**) cells treated with indicated concentrations of SR0987 and LYC55716 for 72 h.

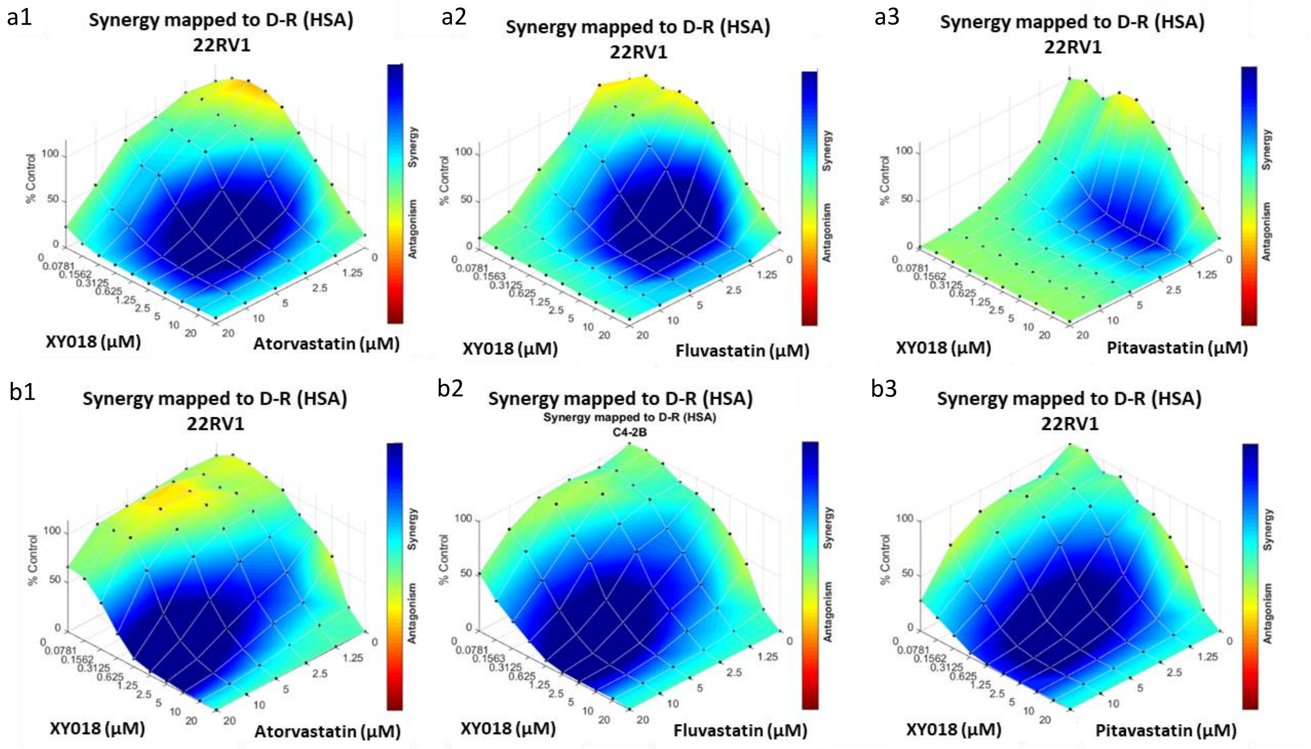

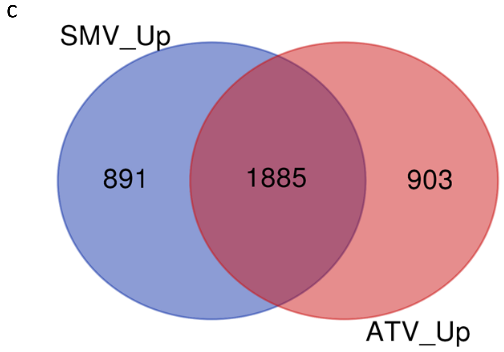

d

Description	LogP
Cholesterol biosynthetic process	-13.39
Sterol biosynthetic process	-3.68
Positive regulation of cholesterol esterification	-3.62
Regulation of lipid metabolic process	-3.37
Lipid metabolic process	-2.68
Isoprenoid biosynthetic process	-2.01

Figure S3. (**a and b**). RORγ antagonists possess synergism with statins in killing mCRPC cells. Drug combination synergism maps of 22Rv1 (**a 1-3**) and C4-2B (**b 1-3**) cells treated with XY018 and ATV, FLV, or PTV at indicated concentrations for 4 days. Blue indicates synergy while red indicates antagonism between drugs. (**c and d**). Statins induced feedback promotes cholesterol biosynthesis gene program expression. Venn diagram of the number of genes with expression significantly upregulated (1.4-fold) in 22Rv1 treated with SMV (5 μM) and ATV (5 μM) for 48 h, as detected by RNA-seq (**c**). Gene ontology analysis of the 1885 genes with expression upregulated by both SMV and ATV in 22Rv1 cells (**d**).

Table S1. Primers and their sequences used in qRT-PCR

Cholesterol Biosynthesis	Sequence	Cholesterol Efflux	Sequence
ACAT2_F	GCAGGTGTTCCTTCAATGGT	NR1H3_F	CAGATCCGCCTGAAGAAACT
ACAT2_R	CACAGCTTTTAGGCCTGACC	NR1H3_R	TTAGCATCCGTGGGAACATC
HMGCS1_F	AGCTCAGAGAGGACACCCAT	NR1H2_F	ACAGCGGCTCAAGAACTAATG
HMGCS1_R	GGTACTTTCTTGGCAGGGCT	NR1H2_R	CGATCTCCTGGACTGAGATGAT
HMGCR_F	CCCAGCCTACAAGTTGGAAA	ABCA1_F	GGAGCTGTTCACCGACAATAAG
HMGCR_R	GCTCCCATCACCAAGGAGTA	ABCA1_R	CCCACCAAGTCCCAAGATAATG
MVK_F	GCTCAAGTTCCCAGAGATCG	ABCG1_F	GGTGGTCTCGCTGATGAAA
MVK_R	ATGGTGCTGGTTCATGTCAA	ABCG1_R	AATCTGCTGGGTTGTGGTAG
PMVK_F	CGGAGAGTGTCTGACATCCA	MYLIP_F	GTGGAGCCTCATCTCATCTTAC
PMVK_R	AAGTTGTCCAGGCCACATTC	MYLIP_R	GCACAGAGCTCCTCATAGTTATAC
MVD_F	AGGACAGCAACCAGTTCCAC	APOA5_F	GAAAGGTGTGGGCTGTGATA
MVD_R	GTGTCGTCCAGGGTGAAGAT	APOA5_R	AAGTCTAGGCTTCAACTTGGG
IDI1_F	TGTTCCCTGCGAAAGGTATC	APOE_F	TCACAGGCAGGAAGATGAAG
IDI1_R	TGAACCTGTTGCTTGTCGAG	APOE_R	AGCGCAGGTAATCCCAAA
GGPS_F	ACACGGTGAAACCCTGTCTC	LRP1_F	GATAGCCAACTCCCAGAACATC
GGPS_R	AGAGGCACTATCTCGGCTCA	LRP1_R	CTCATCCACGAAGCCCTTTAG
FDPS_F	AGGGCAATGTGGATCTTGTC	NPC2_F	CCGAGCTTGGAACTTCGTTAT
FDPS_R	GAAAGAACTCCCCCATCTCC	NPC2_R	GGTGACATTGACGCTGTAAGA
FDFT1_F	GGTCCCGCTGTTACACAACT		
FDFT1_R	AAAACTCTGCCATCCCAATG		
SQLE_F	GGCCATCTTTTGTTGGAGAA		
SQLE_R	TTCAGAAGGGAATGGGAGTG		
LSS_F	TTCCTGAGGCTCTCACAGGT		
LSS_R	CCCTCCATCTGGATTTCTCA		
CYP51A1_F	GCTCAGTTGTTCCCTGCTTC		
CYP51A1_R	AAAATTAGCCAGGCATGGTG		
TM7SF_F	CGCTTTCATCTTCAGCCTCT		
TM7SF_R	GCTCTGCCTCCTTCATCAAC		
MSMO1_F	ACTCTGTCTCCTTGGCTGGA		
MSMO1_R	CATCGTGAAACCCCATCTCT		
NSDHL_F	CTCAGCCAGTCACTCCTTCC		
NSDHL_R	CTGCCTGCTTCAAGAAATCC		
HSD17B7_F	CGTAGGACTTCCGAAAGCAG		
HSD17B7_R	AGACAGCTTCTGCCTTGCTC		
EBP_F	GCCTCAGCACCTAAGACTGG		
EBP_R	ATGAACCCACACACTGCAAA		
SC5D_F	TATCTCTTCCGCCCATGTTC		
SC5D_R	TGGCTCATTCACCATTTCAA		
DHCR7_F	CATTGACATCTGCCATGACC		
DHCR7_R	ACAGGTCCTTCTGGTGGTTG		
DHCR24_F	TGTTGCCTGAGCTTGATGAC		
DHCR24_R	GACCAGGGTACGGCATAGAA		

Table S2. Antibodies used in immunoblotting

Antibody	Vendor	Catalog #	Host Species
ACAT2	Abcam	ab131215	Rabbit
HMGCS1	Santa Cruz Biotechnology	sc-166763	Mouse
HMGCR	Santa Cruz Biotechnology	sc-271595	Mouse
MVK	Proteintech	12228-1-AP	Rabbit
SQLE	Santa Cruz Biotechnology	sc-271651	Mouse
DHCR24	Santa Cruz Biotechnology	sc-398938	Mouse
LXRα	Active Motif, Inc.	61175	Rabbit
LXRβ	Active Motif, Inc.	61177	Rabbit
ABCA1	Santa Cruz Biotechnology	sc-58219	Mouse
ABCG1	Proteintech	13578-1-AP	Rabbit
GAPDH	Cell Signaling Technology	14C10	Rabbit
